# Receptor-interacting protein 1 kinase inhibition therapeutically ameliorates experimental T cell-dependent colitis in mice

**DOI:** 10.1038/s41419-020-2423-2

**Published:** 2020-04-06

**Authors:** Thomas Gobbetti, Scott B. Berger, Kathryn Fountain, Tom Slocombe, Alison Rowles, Gail Pearse, Isobel Harada, John Bertin, Andrea C. Haynes, Allison M. Beal

**Affiliations:** 1 Adaptive Immunity Research Unit, GlaxoSmithKline, Stevenage, Hertfordshire UK; 2 Innate Immunity Research Unit, GlaxoSmithKline, Collegeville, PA USA; 3 Department of Pathology, GlaxoSmithKline, Ware, Hertfordshire UK; 4 DMPK, GlaxoSmithKline, Stevenage, Hertfordshire UK

**Keywords:** Pharmacodynamics, Chronic inflammation

Receptor-interacting protein 1 (RIP1) has emerged as a key protein for transducing signals induced by several immune receptors, including tumour necrosis factor receptor 1 (TNFR 1). When these receptors are engaged, RIP1 kinase activity can drive cell death (apoptosis and necroptosis) and proinflammatory cytokine production which has been implicated in the pathogenesis of multiple inflammatory diseases^1,2^. To date, although RIP1 kinase has been implicated in driving inflammation in multiple tissues, the data points to a key role of RIP1 in the intestines.

Inflammatory bowel disease (IBD) is a multifactorial disorder characterised by chronic intestinal inflammation and comprises two distinct diseases: ulcerative colitis (UC) and Crohn’s disease (CD). Human mutations in the RIP1 pathway, as shown in patients carrying a deficiency in NF-κB essential modulator (NEMO) or the linear ubiquitin chain assembly complex (LUBAC), result in TNF-dependent intestinal inflammation^3,4^. Similar to the human data, NEMO^IEC-KO^ mice display Paneth cell apoptosis and microbiota-driven chronic inflammation in the colon^5^. Additionally, it has been shown that SHARPIN-deficient cpdm mice, that have a defect in LUBAC, develop TNF-dependent multiorgan inflammation^6^. Moreover, RIP1 might be a key driver of Paneth cell death as suggested by increased necroptosis in human Paneth cells in the terminal ileum of patients with CD^7^. It has also recently been shown that necroptosis is strongly associated with intestinal inflammation in children with IBD and that it contributes to strengthening the inflammatory process^8^. Together, these data suggest that the inhibition of RIP1 kinase activity represents an attractive therapeutic target for TNF-driven IBD in an integrated system.

At GlaxoSmithKline (GSK) we have developed small molecule inhibitors which potently bind to RIP1 with exquisite kinase selectivity and excellent activity in protecting from hypothermia in a model of TNF-induced sterile shock^9^. Furthermore, these molecules have also been shown to reduce the spontaneous production of cytokines from human UC and CD explants^10^. However, the use of animal studies is also critical in addressing mechanistic and translational questions related to IBD.

A recent study showed that necrostatin-1 (Nec-1) reduced intestinal inflammation in the chemically-induced DSS model of colitis^11^. Nec-1 is a tool used to explore the function of RIP1 kinase activity. However, its utility is limited due to the moderate potency, off-target activity against indoleamine-2,3-dioxygenase (IDO), and poor pharmacokinetic properties. GSK547 is a potent and highly-selective inhibitor of RIP1 kinase activity with suitable pharmacokinetic properties for dosing in chronic mouse models. In this study, we have tested for the first time the anti-inflammatory potency of GSK547 in the CD4^+^CD45RB^high^ T cells transfer mouse model of colitis. Among all models of colitis, the T cell transfer model is significantly more relevant to human disease to investigate the immunological mechanisms responsible for the induction and perpetuation of chronic intestinal disease. This model has proved the most reliable in predicting the efficacy of therapies that have successfully progressed into the clinic (i.e. anti-IL-12/23 p40 monoclonal antibody therapy)^12^. Moreover, when compared with other animal models, the CD4^+^CD45RB^high^ T cells transfer model shows the most resemblance to human IBD in terms of colon gene expression changes^13^.

In this study, chronic colitis was achieved by transferring CD4^+^CD45RB^high^ T cells into immunodeficient female SCID mice (Supplementary Materials and Methods). After confirming the development of pathology using endoscopy (Supplementary Fig. 1), animals were treated therapeutically with GSK547 (50 mg/kg) or vehicle (0.5% hydroxypropyl methylcellulose in water twice a day per os) starting at 3 weeks after cell transfer (*n* = 10/13 mice per group). This dose was selected to maintain coverage of IC90 for 12 h based on the integration of known pharmacokinetic profiles and relevant in vitro potency (Supplementary Fig. 2). The severity of colitis was measured at termination (day 35) (Fig. 1a). The most common in-life indicator for this autoimmune colitis is body weight loss. Our data show that treatment with GSK547 significantly prevented body weight loss when compared with the vehicle treated group (Fig. 1b). Post-mortem pathological observations showed that the treatment significantly ameliorated experimental T cell-dependent colitis in mice. GSK547-treated mice displayed decreased colon density (ratio weight/length), macroscopic signs of inflammation (disease activity index—scores of oedema, diarrhoea, presence of blood in the stools) and colon thickness (Fig. 1c–e). In clinical IBD, endoscopic and histopathologic indices are routinely used as outcome measures in controlled clinical trials and are essential for both fundamental and translational research in mouse models. Mucosal damage, assessed both by endoscopy and histology, was significantly reduced following treatment with the RIP1 kinase inhibitor (Fig. 1f, g). It is known that T cell driven colitis is generally associated with increased expression of tissue cytokines. In this study, the reduced protein expression of IFN-γ, IL-17A, TNF-α, CXCL-1, IL-6 and IL-12/23p40 in the colon (Fig. 1h–m), suggests that Type 1 (Th1) and Type 17 (Th17) helper responses were suppressed after treatment with GSK547. High-circulating serum amyloid A^14^ and faecal calprotectin^15^ levels are widely used markers in IBD for evaluating intestinal inflammation and mucosal healing. Both of these translational biomarkers were also decreased in the GSK547-treated group compared with vehicle controls, indicating the potential for RIP1 inhibitors to decrease markers of systemic inflammation and neutrophilic infiltration in the colon (Fig. 1n, o).

**Fig. 1:**
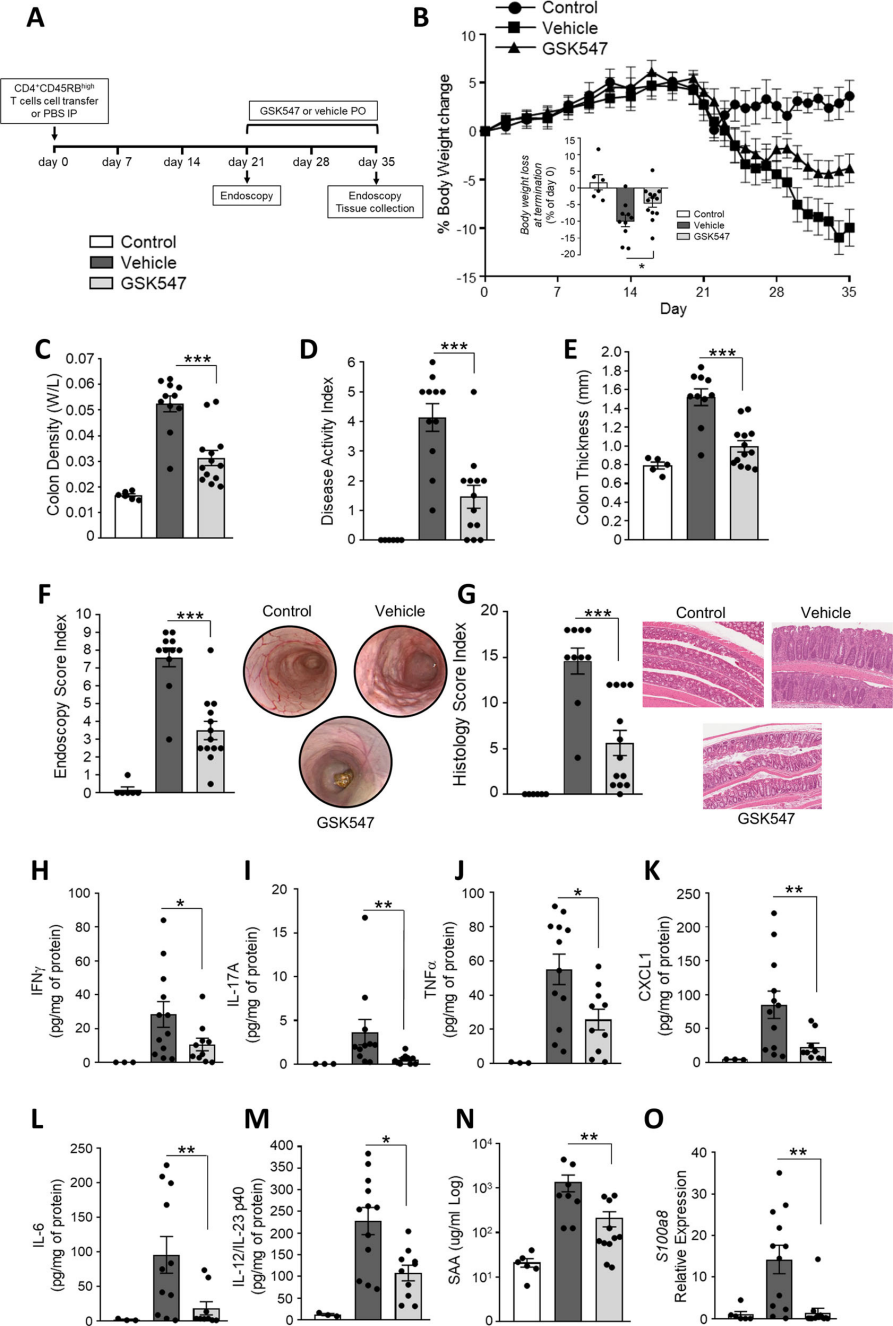
RIP1 kinase inhibition suppresses inflammation in T cell transfer model of colitis. Animals received food and water ad libitum. All animal studies were ethically reviewed and carried out in accordance with Animals (Scientific Procedures) Act 1986 and the GSK Policy on the Care, Welfare and Treatment of Animals. **a** Experimental outline. Female SCID mice (6–8 week-old) received CD4^+^CD45RB^high^ T cells from BALB/c mice (*n* = 10/13 mice per group) or PBS via intraperitoneal injection (*n* = 6 mice). After confirming the development of pathology using endoscopy, animals were treated therapeutically with the GSK547 (50 mg/kg twice a day per os) or vehicle (0.5% hydroxypropyl methylcellulose in water). **b** Body weight loss compared to starting weight. **c** Colon density (weight:length). **d** Disease activity index (scores of oedema, diarrhoea, presence of blood in the stool) was used to determine the clinical outcome of colitis on the day of sacrifice. **e** Colon thickness measured using calipers. **f** Endoscopy score based on the assessment of thickening, vasculature, granularity of intestinal mucosa. **g** Histology score based on epithelial hyperplasia, mucosal inflammatory cell infiltrate and the extent of the lesions. **h**–**m** Multiplex measurement of proinflammatory cytokines in mouse colon measured by MSD. **n** Plasma SAA levels measured by ELISA. **o** Intestinal *S100a8* levels measured by RT-PCR. The experiment was conducted in two independent blocks, commenced on successive days. Data presented as means ± SEM. For pair-wise comparison of means, ***p < 0.001, **p < 0.01, *p < 0.05. Statistical analysis was performed using a linear mixed model with block as a random effect, using the software JMP, Version 14.2.0 (SAS Institute Inc.).

Taken together, our results suggest that RIP1 kinase inhibition has anti-inflammatory potential which strongly protects against the progression of chronic colitis. The efficacy following therapeutic administration of a potent and selective RIP1 kinase inhibitor on multiple endpoints in a translatable autoimmune model of colitis provides further scientific insight to support the rationale for an existing novel target in the management of IBD. These findings specifically align to the ongoing clinical programme evaluating the effect of RIP1 kinase inhibition in IBD patients.

## Supplementary information

Supplementary Figure 1

Supplementary Figure 2

Supplementary Materials and Methods
