# Supplementary material for: Receptor-interacting protein 1 kinase inhibition therapeutically ameliorates experimental T cell-dependent colitis in mice
Source: Cell Death Dis. 2020 Apr 6;11(4):220. doi: 10.1038/s41419-020-2423-2 (PMC7136199; doi:10.1038/s41419-020-2423-2)
Supplement: Supplementary file 1 — Supplementary Figure 1 [file 41419_2020_2423_MOESM1_ESM.docx]

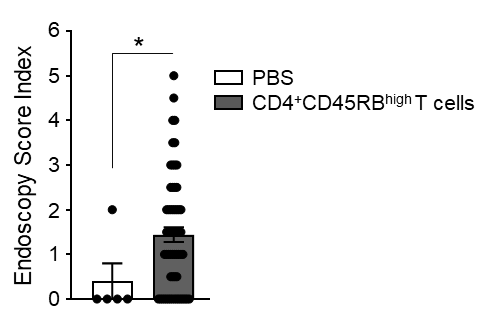


**Supplementary Figure 1**

Endoscopy score at day 19 in female SCID mice injected with CD4^+^CD45RB^high^ T cells from BALB/c mice or PBS via intraperitoneal injection.
