# Supplementary material for: Receptor-interacting protein 1 kinase inhibition therapeutically ameliorates experimental T cell-dependent colitis in mice
Source: Cell Death Dis. 2020 Apr 6;11(4):220. doi: 10.1038/s41419-020-2423-2 (PMC7136199; doi:10.1038/s41419-020-2423-2)
Supplement: Supplementary file 2 — Supplementary Figure 2 [file 41419_2020_2423_MOESM2_ESM.docx]

**
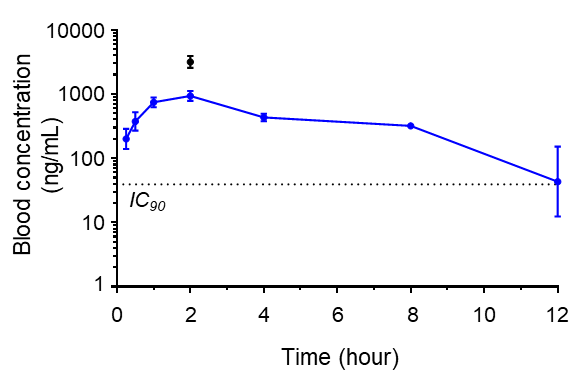
**

**Supplementary Figure 2**

In blue, concentrations of GSK547 in single dose pharmacokinetic study. N=3 mice per time point were bled via tail vein over 12 hours following 50 mg/kg PO administration. In black, GSK547 concentrations measured 2 hours following compound administration in the T cell transfer model of colitis at day 21. All values represent geometric mean and standard deviation. The horizontal line represents the IC_90_ concentration calculated using the potency from the L929 *in vitro* assay (total blood IC_50_=12.5 ng/mL, hillslope = 1.74)^1^.

1 Wang, W. *et al.* RIP1 Kinase Drives Macrophage-Mediated Adaptive Immune Tolerance in Pancreatic Cancer. *Cancer Cell* **34**, 757-774 e757, doi:10.1016/j.ccell.2018.10.006 (2018).
