# Supplementary material for: Receptor-interacting protein 1 kinase inhibition therapeutically ameliorates experimental T cell-dependent colitis in mice
Source: Cell Death Dis. 2020 Apr 6;11(4):220. doi: 10.1038/s41419-020-2423-2 (PMC7136199; doi:10.1038/s41419-020-2423-2)
Supplement: Supplementary file 3 — Supplementary Materials and Methods [file 41419_2020_2423_MOESM3_ESM.docx]

**Supplementary Materials & Methods**

**Mouse experiment**

C.B-17 SCID mice and BALB/c mice were purchased from Envigo US and Charles River UK. Animals received food and water ad libitum. All animal studies were ethically reviewed and carried out in accordance with Animals (Scientific Procedures) Act 1986 and the GSK Policy on the Care, Welfare and Treatment of Animals.

The T-cell transfer model was performed as previously described^1^. Briefly, leukocytes were isolated by passing spleens from BALB/c donor mice through a 70µM strainer. Red blood cells were removed by incubation in RBC lysis buffer (eBioscience, San Diego, CA, USA). CD4 T cells were enriched using a Mouse CD4 T Cell Isolation Kit (Miltenyi Biotec, Bergisch Gladbach, Germany), according to manufacturer’s instructions. Resulting untouched cells were stained with anti-mouse CD4-APC and CD45RB-PE (Biolegend, San Diego, CA, USA) for 30 minutes on ice. Naïve CD4 T cells were further purified by sorting on a FACSAria III Fusion (Becton Dickinson, Franklin Lakes, NJ, USA), by gating on the CD4+ CD45Rb^high^ fraction. CD45RB^high^ fraction was defined as the 40% brightest cells within the CD4+ population. Mice were randomly assigned to experimental groups. Colitis was induced by injecting 3×10^5^ CD4^+^CD45RB^high^ T cells intraperitoneally. Control animals were injected with PBS only. After confirming development of pathology using endoscopy at day 19, animals were treated therapeutically with the GSK547 (50 mg/kg twice a day per os) or vehicle (0.5% hydroxypropyl methylcellulose in water) from day 21 to day 35. At day 21 mice were bled 2 hours following dosing of GSK547 or vehicle via tail vein. At the end of the study blood was obtained by cardiac puncture and plasma stored at −80°C. Colon was weighed and measured for length (Colon density being the ratio weight/length). Disease activity index (scores of oedema, diarrhoea, presence of blood in the stool) was used to determine the clinical outcome of colitis on the day of sacrifice. Colon thickness was measured using callipers. The Endoscopy Score Index, based on the assessment of thickening, vasculature, granularity of intestinal mucosa, and the Histology Score Index, based on epithelial hyperplasia, mucosal inflammatory cell infiltrate and the extent of the lesions, were determined as previously described^2^. For both the histological slides and endoscopy videos, readers were blinded to the experimental group. Sections of colon were removed, immediately frozen in and stored at −80°C for cytokine and gene analysis.

**Plasma Serum amyloid A**

Serum amyloid A (SAA) concentrations were analysed in plasma by using Phase Range Mouse SAA Elisa kit (Tridelta Devlopment Ltd., Maynooth, Ireland).

**Intestinal tissue homogenates and MSD analysis**

Colon tissue was weighed and homogenized in Tris lysis buffer (Meso Scale Diagnostics Ltd., Rockville, MD, USA) with protease inhibitors (Roche, Woerden, The Netherlands) using Precellys tissue homogenizer tubes (Bertin Technologies, France) for 6000 rpm, twice x 10 seconds. Afterwards, samples were spun down for 5 minutes at 13000 rpm at 4°C and the supernatant was transferred to a clean tube. Protein concentrations were determined by BCA kit (ThermoFischer Scientific, Waltham, MA, USA). Intestinal homogenates were analysed for the presence of IFNγ, TNF-α, IL-6, IL-12/23 p40, IL-17A, and CXCL1 by MSD according to manufacturer’s instructions.

**Quantitative RT-PCR**

mRNA was extracted from mouse colons with the Rneasy Mini Kit (Qiagen, Germantown, MD, USA) according to manufacturer’s conditions. Complementary DNA was synthesized from mRNA using SuperScript IV VILO Master Mix with ezDNase (ThermoFisher Scientific). Quantitative RT-PCR was performed on a AB Quantstudio 7Flex Taqman (Thermofisher Scientific)  using Taqman Fast Advanced Master Mix and mouse specific oligonucleotide S100a8 (Mm00496696_g1 - Calprotectin subunit). mRNA levels were normalised against *Eif2b1* and *Gusb* and gene expression was calculated with the 2^-ΔΔct^ method.

**Pharmacokinetic Analysis**

For dose selection, a pharmacokinetic study was conducted following a single oral dose of GSK547 (50 mg/kg in 0.5% hydroxypropyl methylcellulose in water) and blood samples were collected at various times for drug level determination. Pharmacokinetic samples were also collected at a single timepoint (2 hours post dose of GSK547 at day 21) in the T cell transfer study and diluted with an equal volume of water. These blood samples were collected to confirm compound administration and to compare to blood concentrations measured in a previous pharmacokinetic study. Compound was extracted by protein precipitation by adding 9 volumes of acetonitrile. Samples and standards were quantified by LC-MS/MS. Chromatography was performed at 40°C using the Phenomenex KintexEvo C18 2.6 um 50*2.1 mm column (Phenomenex, Macclesfield, UK). Mass Spectrometry was performed using the API 4000 Qtrap (Sciex, Warrington UK) equipped with a turbo ion spray source. Drug concentration was calculated using chromatographic peak area using Analyst 1.6.

**References**

1 Read, S. & Powrie, F. Induction of inflammatory bowel disease in immunodeficient mice by depletion of regulatory T cells. *Curr Protoc Immunol* **Chapter 15**, Unit 15 13, doi:10.1002/0471142735.im1513s30 (2001).

2 Koelink, P. J. *et al.* Development of Reliable, Valid and Responsive Scoring Systems for Endoscopy and Histology in Animal Models for Inflammatory Bowel Disease. *J Crohns Colitis* **12**, 794-803, doi:10.1093/ecco-jcc/jjy035 (2018).
